# Newly Hatched Stage I American Lobster (*Homarus americanus*) Survival Following Exposure to Physically and Chemically Dispersed Crude Oil

**DOI:** 10.1007/s00244-022-00912-z

**Published:** 2022-01-27

**Authors:** Benjamin P. de Jourdan, Tahereh Boloori, Les E. Burridge

**Affiliations:** Huntsman Marine Science Centre, Aquatic Biosciences, St. Andrews, NB Canada

## Abstract

Standard model species are commonly used in toxicity tests due to their biological and technical advantages but studying native species increases the specificity and relevance of results generated for the potential risk assessment to an ecosystem. Accounting for intraspecies variability and other factors, such as chemical and physical characterization of test medium, is necessary to develop a reproducible bioassay for toxicity testing with native species. In this study, larval stage I American lobster (*Homarus americanus*), a commercially important and native species of Atlantic Canada, was used as the test species. Toxicity tests were first conducted by exposing lobster larvae to a reference toxicant of copper sulphate (CuSO_4_) and then to physically and chemically (using Corexit 9500A) dispersed oil (WAF and CEWAF, respectively). The effect on larval survival was estimated by calculating the 24-h median effect concentration (24-h EC50), and there was no difference between WAF or CEWAF exposure when the results are reported on a total petroleum hydrocarbon (TPH) basis. The 24-h EC50s ranged from 2.54 to 9.73 mg TPH/L when all trials (*n* = 19) are considered together. The HC5 (hazardous concentration for 5 per cent of the population) value was 2.52 mg TPH/L and similar to the EC50 value when all trials were pooled. To evaluate the reproducibility of the lobster toxicity tests, inter-trial variability was determined, and the resultant coefficients of variation (%CV) were compared to those reported for two standard test species, mysid shrimp (*Americamysis bahia*) and inland silverside (*Menidia beryillina*). This comparison showed that the %CV for the lobster toxicity tests were lower than those for the standard species tests indicating that the described larval lobster toxicity bioassay produces reliable and repeatable results.

**Supplementary Information:**

The online version contains supplementary material available at 10.1007/s00244-022-00912-z.

A common approach to minimize the impact of biological variability on the outcome of toxicity tests is to rely on laboratory cultures of standard test species, where a reduction, or exclusion, of genetic diversity serves to reduce experimental variability. The standard model species (e.g. *Daphnia magna*, *Oncorhynchus mykiss*, *Danio rerio, Americamysis bahia*, *Menidia beryllina*) generally meet certain technical criteria, such as easy and inexpensive maintenance in the laboratory, genetic tractability, and availability of a broad spectrum of experimental and methodological tools (Segner and Baumann 2015). These species also meet some biological criteria, such as fast growth, high reproductive potential, sensitivity to pollutants, abundance, replenishment ability, and/or captive rearing capabilities (Hughes et al. 2005). These practical and regulatory testing requirements of tests species have focused more research on testing a limited number of species to provide data from which comparison of relative toxicity of chemicals can be made. Extrapolations are also often made to predict the toxicological and ecological responses of other related species in the ecosystem. These extrapolations are typically based on descriptive or statistical relationships, such as uncertainty, safety, or assessment factors or interspecies correlation estimates (Bejarano and Barron 2014). The use of indigenous or native species, rather than a limited number of standard species, will increase the specificity and relevance of the data generated. However, there are often inherent problems working with these species, such as limited availability, lack of requisite information necessary for their acclimation and maintenance under laboratory conditions, and insufficient background information on their sensitivity and the reproducibility in toxicity testing (Echols et al. 2015). Regardless, as part of a Natural Resource Damage Assessment (NRDA), it is necessary to try and determine the effects of exposure on local, native species (Echols et al. 2015). This may be achieved by an aquatic toxicity programme that uses field collected and non-standard test organisms of local relevance and importance. However, it is necessary to validate that the results gained from bioassays are precise and reproducible for these species to be successful in a toxicity programme. A possible significant factor contributing to the reproducibility may be the intraspecific variation that could contribute to either over- or underestimating toxicity. Understanding the intraspecies variability and test precision of a proposed bioassay with a native species will allow the data to be properly interpreted and used for risk and damage assessments.

American lobster (*Homarus americanus*) is not a standard test species; however, due to its economic and ecological importance in eastern North America, it is prudent to characterize its risk from exposure to environmental contaminants, such as those associated with an oil spill. The first larval stages (I–III) of the American lobster are truly pelagic and considered to be more sensitive to contaminants and other stressors than the later benthic and adult stages (Burridge and Haya 1997). Hatching of larval lobsters occurs over a period of two or more months beginning in early to late June in the central and southern coastal Gulf of Maine, and about a month later in southern Nova Scotia and Browns Bank (Incze and Naimie 2000). The duration of the planktonic larval phase (stages I-III, and one postlarval stage, stage IV) is temperature dependent and may vary between laboratory reared and field organisms. Annis et al. (2007) reviewed and modelled larval development times for *H. americanus* and noted regional variations in field developmental times between the peak abundance of stage I and postlarvae, ranging from 12 days in the Gulf of Maine to up to 35 days off the Magdalen Islands (Gulf of St. Lawrence). These pelagic stages also represent the greatest potential for exposure to hydrocarbons associated with an oil spill and spill response measures (e.g. application of chemical dispersants), which tend to be more concentrated in surface waters.

The toxicological responses of American lobster exposed to petroleum hydrocarbons are less well understood compared to other crustaceans, such as the mysid shrimp (*Americamysis bahia*) a standard marine test organism. The sensitivity of pelagic lobster larvae (i.e. stages I–III) to crude oil has been previously demonstrated in laboratory studies (Wells and Sprague 1976) and as a result of accidental spills in the field (Reddy and Quinn 2001). The 1996 grounding of the *North Cape* barge off the southwestern coast of Rhode Island (USA) resulted in an estimated 2700 metric tons of fuel oil entering into the shallow near shore waters, killing approximately 9 million lobsters (French-McCay 2003).

Species sensitivity in response to a stressor may be variable due to experimental and natural parameters that need to be considered when investigating the impact of hazardous compounds to aquatic species. This data variability from laboratory-based toxicity tests introduces uncertainty when extrapolating for environmental risk assessment purposes. Thus, it is crucial to properly characterize the experimental and natural variabilities that may influence toxicity test results (Simmons et al. 2015). Mayer and Ellersieck (1986) demonstrated that physical conditions of the test environment, such as pH and temperature, and chemical properties of the test compound, such as solubility, can significantly influence toxicity test results. Biological variability proves more challenging to address as there are multiple contributing sources, including seasonal and temporal variation, genetic variation amongst individuals, and choice and life stage of test species (Hrovat et al. 2009; Simmons et al. 2015), differences in physiological status (e.g. size, age, sexual maturity status, etc.), and differential sensitivities of individuals to natural environmental parameters (Devin et al. 2014). Assessing the inter-individual variability of American lobster larvae is crucial for understanding its utility as a model test organism and understanding the risks to the population. Having more complete knowledge about the sensitivity of lobster larvae to petroleum hydrocarbons will also allow for a comparison with standard test species to better understand the relative sensitivity of this non-standard, commercially important species.

In the present study, we investigated the utility of American lobster larvae as a native test species in aquatic toxicology studies. Following method development and reference toxicant testing, the bioassay was used to assess the toxicity of petroleum hydrocarbons and the variability in response amongst different batches of larvae from the same lobster and between different lobsters. We hypothesized that there would not be a difference in response amongst larval batches from the same lobster, nor would there be differences between batches from different lobsters.

## Methods

### Test Organism: American lobster (Homarus americanus)

This study was undertaken using the first planktonic life stage (i.e. stage I) of the American lobster. Under a Fisheries and Oceans Canada special permit, adult commercial-size (0.5–2.0 kg) ovigerous (“berried”) females were collected from the Bay of Fundy Lobster Fish Area 36 by local fishers. The lobsters (*n* = 30, given sequential lobster IDs of 1–30) were transferred to the Huntsman Marine Science Centre (St. Andrews, NB) where they were held under controlled environmental conditions to promote egg mass development. Berried lobsters were transferred to an individual holding tank that received seawater of 18 °C ± 2 °C when visual inspection of embryo development indicated that larval release was imminent. Larval lobsters released from each overnight hatch (termed a batch) were separately collected and held for single batch toxicity tests. Twenty lobster larvae were collected from each batch then imaged as reference organisms using a Leica Wild M420 microscope and Leica MD190 camera at 12.5 × magnification. Carapace length (length from the rear of the orbital socket to the rear edge of the carapace) of reference lobster larvae was measured using the LAS software version 4.8. Weight and carapace length of berried female lobsters were also recorded to examine the role of maternal, morphological traits in explaining variability in response. A total of 14 of the collected 30 lobsters were utilized in this study.

### Exposure Methodology

**Fig. 1:**
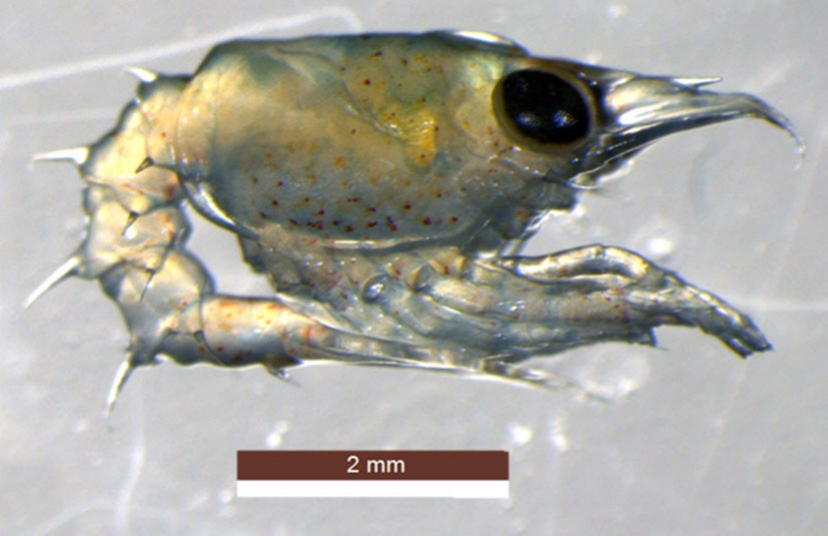
Stage I larval lobster (Homarus americanus) at 24-h post hatching

For American lobster, a classic breeding colony is not maintained as contributing adults are collected annually from new wild stock. The bioassay methodology was modified from previous internal studies performed at the Huntsman Marine Science Centre. Briefly, larval lobsters were collected from a single 24-h release from a single female lobster and transferred into an environmental chamber (temperature maintained at 15 °C  ± 2 °C and a photoperiod of 16 h light and 8 h dark). The transfer bucket was vigorously aerated to minimize cannibalism within the population. From the transfer bucket, a subsample of larvae was collected in a 600 mL beaker and placed on a light table (A4 LED Light Box, FOME). An individual larva that was actively swimming in the water column was retrieved from the beaker using a transfer pipette modified to have a large, smooth opening and dispensed onto the corner of a folded KimWipe to remove excess water then visually confirmed to be stage I larvae as characterized by the absence of pleopods (Fig. 1).

The larva was gently transferred into a 25 mL scintillation vial filled with 20 mL of test media (allowing 20% headspace for surface oxygen exchange) using a metal scoopula and observed for 30 s to ensure larval viability and health before capping the vial (screwed tight, then loosened by a ¼ turn). There was no renewal of test solution during the exposure. The larvae were exposed with 1 individual per experimental unit to prevent cannibalism, with 10 replicate units per concentration. The larvae were observed during and after the exposure for 30 s and scored using the numeric categories described in Table 1.

**Table 1:** Assessment endpoint scoring criteria for lobster larvae

Score	Description
0	No observed effect: vigorously swimming, active internal organ movement
1	Affected: passive swimming, erratic swimming, positioned on side or back, rigid body position, exopodites/pereiopod beating in coordinated motion
2	Moribund (mortally affected): no swimming activity, twitching, sporadic movement of mouthparts and exopodites/pereiopod, positioned on side or back, faint heartbeat, slight internal organ movement
3	Dead: no swimming, no visible heartbeat, change in coloration towards brown/opaqueness, absence of movement after gentle prodding

Larvae were considered immobilized if they had a score of 2 or greater, and inline with other invertebrate methods, immobilization is considered here as a surrogate for death (USEPA 2016). Effect concentrations were calculated based on immobilization (EC50) and lethality (LC50) at 24 and/or 48 h. Validity criteria for the toxicity tests included standard water quality-based measures (i.e. greater than 60% dissolved oxygen saturation and less than 1.5 °C variation of temperature amongst treatment vials), as well as a species specific control survival criterion of less than 20% mortality, based on background studies and relatively high mortality rates for the species in an aquaculture environment (Sprague and McLeese 1968).

## Reference Toxicant Testing

The availability of the larval lobster is seasonally limited (late summer, early fall). As such, there is only a short window of opportunity for toxicity testing, and a year-round reference toxicant testing programme is not feasible. Despite this limitation, the same reference toxicity testing principles were applied to the lobster larvae during their seasonal availability. Copper sulphate was selected as the reference toxicant given its desirable traits (e.g. established toxicity database, readily available, water soluble, stable, easily analysed) and efficacy in providing a consistent and measurable effect in larval lobsters. A total of four reference toxicant tests were completed with concentrations of 0, 3.3, 10, 33, 100, 330, and 1000 µg Cu/mL, and larvae were scored at 24 and 48 h.

### Preparation of WAF and CEWAF Stock Solutions

Exposure waters were prepared in an environmental chamber (15 °C  ± 2 °C) following a benchtop mixing method that modified the baffled flask method for dispersant effectiveness used by Environment Canada and the United States Environmental Protection Agency (Venosa et al. 2002) and the CROSERF method (Singer et al. 2000). Briefly, 1.6 L of 0.22 µm filtered seawater from the Bay of Fundy was poured into a clean 2 L baffled flask. Water accommodated fractions (WAFs) were prepared by dispensing crude oil (artificially weathered by nitrogen stripping until 10% loss by mass) from offshore Newfoundland and Labrador onto the central surface of the water in the flask at a loading of 1 g oil/L of water. Chemically enhanced water accommodated fractions (CEWAFs) were prepared by adding Corexit 9500A to the centre of the surface oil slick at a dispersant to oil ratio of 1:20. The mixing flask was then sealed with DuraSeal, secured on an orbital shaker (MaxQ SHKE2000 digital shaker, Thermo Scientific), and shaken at 150 rpm for 1 h before allowed to settle for 1 h. This mixing method was calibrated to mimic droplet size and distribution results obtained from a wave tank study conducted by SL Ross (Ottawa, ON) that served as a proxy for offshore Newfoundland and Labrador conditions. These stock solutions were then diluted to generate exposure media. A dispersant only control was prepared at a concentration that was equal to the volume of dispersant in the highest tested CEWAF concentration and was nominally 15.8 mg/L in the range finding test (equal to the highest CEWAF concentration tested, 32%) and 4.75 mg/L in the definitive tests (equal to the highest CEWAF concentration test, 10%).

### Acute Toxicity Test with WAF and CEWAF

A total of 19 toxicity tests were conducted between June and September 2018 using 14 female lobsters (see SI Table 1 for additional details). The toxicity tests were conducted in the same manner as described for the reference toxicant testing, with a 24-h exposure duration. The dilution water for toxicity tests was the same filtered seawater that was used to prepare WAF and CEWAF stock solutions. The preliminary range finding trials included test solutions of 100%, 56%, 32%, 18%, and 10% of WAF stock solution and 32%, 18%, 10%, 5.6%, and 3.2% of CEWAF stock solution, as well as a dispersant only control (15.8 mg/L) and a seawater control. The definitive toxicity tests followed with nominal concentrations of 100%, 56% and 32% WAF and 10%, 3.2% and 1% CEWAF stock solutions, along with the dispersant only control (4.75 mg/L) and seawater control, each tested with 10 organisms per treatment. A randomized experimental setup was employed, and treatments were blinded to assessors to eliminate potential bias.

### Exposure Media Characterization

Water quality parameters were measured in three replicates per test solution pre- and post-exposure. Dissolved oxygen (DO%) and temperature (°C) were measured using a YSI model ProSolo Digital Water Quality Meter (Yellow Springs Instruments, Yellow Springs, OH, USA). Salinity (psu) and pH were measured using the YSI model MultiLab 4010–2 (Yellow Springs Instruments, Yellow Springs, OH, USA) calibrated with standard pH buffer solutions.

Samples of each test solution from a single trial were collected at the beginning of the study and sent to a contract laboratory (RPC, Fredericton, New Brunswick) for characterization of total petroleum hydrocarbons (TPH), polycyclic aromatic hydrocarbons (PAHs) and alkyl-PAHs by GC–MS based on the method described in the USEPA 3510C/8270C document (Edgell and Wesselman 1989).

A LISST-100X particle size analyzer (Sequoia Scientific, Inc) was used to characterize the mean droplet concentration value per bin class (VC, μL/L), the particle size distribution, and the mean droplet size calculated from 60 independent measurements of each test solution of every WAF and CEWAF preparation. The concentrations of TPH for the CEWAF were estimated in the rest of the experimental trials based on the regression relationship between VC and TPH. The measured concentrations were used to model immobilization response and determine the effect concentration to 50% of the population (i.e. EC50).

### Statistical Design and Data Analysis

Water quality parameters, carapace length of reference lobsters, and the wet weight of female lobsters were all tested for normality and homogeneity of variance using Shapiro–Wilk and Levene’s tests, respectively. Analysis of variance (ANOVA) using R program was performed to investigate any significant differences within and between datasets. The significance criterion was set at *p* < 0.05. In cases with significant differences, the post hoc Tukey test was performed to find the trial(s) with significantly different effect concentration(s). If the assumptions of normality and homogeneity of variance were not met, then the non-parametric test of Mann–Whitney U (also called Wilcoxon Rank Sum Test) was performed.

The *drc* package (v 3.0–1; Ritz et al. 2015) in R (v 3.6.1; R Core Team 2019) was used to fit the appropriate model to the acute toxicity results. The best-fit model with the smallest Akaike information criterion (AIC) value was selected to represent the concentration–response curve for each experimental trial and used to calculate the 24-h EC50s for the WAF and CEWAF exposures, and a 48-h EC50 for the reference toxicity testing. The variability in the toxic responses of larval lobsters was assessed as a function of maternal origin. In cases where a single female produced more than one batch of larvae, the responses of individual batches were compared to one another. In addition, the responses of larvae from different females were compared across trials. The precision of acute toxicity results for both within and amongst females was evaluated using the coefficient of variation (CV) and standard deviation.

A species sensitivity distribution (SSD) type approach was used with the cumulative distribution of EC50 values to assess the potential risk to the lobster larvae population. A log-normal distribution function and resampled randomly 1000 times were used to generate the SSD mean value and the 5^th^ percentile hazard concentration (HC5) with corresponding 95% confidence interval (95% CI).

The reference toxicant results were compared with literature values curated from the US Environmental Protection Agency ECOTOX database (USEPA, 2019) to assess how the results from this bioassay are compared to previously published values for lobster and other marine crustaceans. To compare the consistency of results and the variability of the bioassay, the CV was determined for the LC50 results (*n* = 19) determined in this study and was compared to the CV from 91 data points obtained from the USEPA National Contingency Plan (NCP) Product Schedule (USEPA 2021) showing the toxicity of No. 2 Fuel oil as 48-h EC50 (mysid shrimp) and 96-h LC50 (inland silverside) on a TPH basis.

## Results

### Reference Toxicant Testing

The 48-h EC50 values ranged from 105.4 to 151.1 µg/L, with a CV of 15.9%. The 48-h LC50 values align with those from the literature (100–330 µg/L, Connor 1972) for larval lobster (*Homarus gammarus*) and the resulting high degree of precision supports the use of this bioassay to study the effects of other contaminants.

### Exposure Media Characterization

#### Water Quality

No significant differences were observed for temperature, salinity, or pH between experimental trials at both pre- and post-toxicity measurements. The DO (% saturation) in control seawater was significantly different from the 1 (*p* = 0.04) and 3.2% (*p* = 0.01) CEWAF treatments across all trials in the post-exposure measurements. The mean value of DO declined from 93.9% (± 2.3%) to 83.3% (± 8.5%) across all trials between pre- and post-exposure but never dropped below 60% saturation. Salinity values followed ambient conditions and ranged from 29 to 32 PSU over the course of the study. pH ranged between 7.33 and 7.90 (mean = 7.76) in the pre-exposure solutions and 6.78–7.94 (mean = 7.75) in the post-exposure solutions. The variability of temperature in the test solutions was not significantly different between the two measurements with an average temperature of 14.4 °C (± 0.6 °C) and 14.5 °C (± 0.5 °C) at the pre- and post-exposure measurements, respectively. The mean, standard deviation and the coefficient of variation (CV; measure of inter-trial variability) of the water quality parameters across all trials (*n* = 19) are shown in Supplemental Information Table 2.

### Chemical Characterization

The chemical characterization of the TPH analytes for the WAF and CEWAF treatments is shown in Table 2.

**Table 2:** Concentration (mg/L) of the total petroleum hydrocarbon (TPH) of nominal concentrations of physically dispersed oil (32% WAF) and chemically enhanced fraction of oil (1%, 3.2% and 10% CEWAF) dispersed using Corexit 9500A

Analytes	MDL	Control seawater	Corexit 9500A Control (nominal 4.75 mg/L)	32% WAF	1.0% CEWAF	3.2% CEWAF	10% CEWAF
Benzene	0.001	< MDL	< MDL	0.015	0.0005	0.001	0.004
Toluene	0.001	< MDL	< MDL	0.31	0.01	0.03	0.12
Ethylbenzene	0.001	< MDL	< MDL	0.06	0.003	0.009	0.034
Xylenes	0.001	< MDL	< MDL	0.3	0.015	0.049	0.18
VPH C6-C10 (Less BTEX)	0.01	< MDL	< MDL	0.52	0.05	0.14	0.45
EPH > C10—C16	0.05	< MDL	< MDL	0.33	0.49	1.6	5.1
EPH > C16—C21	0.05	< MDL	< MDL	0.07	0.4	1.3	3.8
EPH > C21-C32	0.1	< MDL	< MDL	< MDL	0.06	1.8	5.8
Modified TPH Tier 1	0.1	< MDL	< MDL	0.9	1.5	4.8	15.0
**TPH**		0.00	0.00	1.61	1.57	4.93	15.49

*MDL Method detection limit

Values ≤ MDL were replaced with $\frac{1}{2}$ MDL for calculating sums

### Physical Characterization

The concentrations of each analyte increased as the nominal concentration of the chemically dispersed oil elevated (i.e. 1%, 3.2% and 10%). The measured concentrations of TPH analytes were below the method detection limit (MDL) of the GC–MS in the control seawater and dispersant only control.

In the WAF samples, the LISST transmissivity was too great (> 85%) resulting in an unreliable reading and an instrument warning that the sample was “too clear”. As such, only the results from the CEWAF solutions are presented. The volume concentration (VC) in each bin increased with increasing strength of test solution and was linearly correlated with increase in TPH concentration. There was very good consistency between preparations in terms of droplet profile and concentrations. The mean droplet size from the nine different preparations of 3.2% CEWAF ranged from 3.9 to 10.8 µm with a mean value of 6.3 (standard deviation = 2.4) (SI Fig. 1).

### Bioassay

A total of 19 bioassays were performed using American lobster larvae from 14 individual berried females. There were significant differences observed between the carapace length (mm) of lobster larvae from different batches and/or different females (*p* < 0.05). The carapace length of reference larvae ranged from 1.74 to 2.21 mm with the average length of 1.99 mm (± 0.087 mm, *n* = 380) and increased linearly with increasing adult lobster wet weight (SI Fig. 2).

Immobilization was assessed in each individual larvae following the 24-h exposure. The performance of lobster larvae in the control seawater and the dispersant only control met the validity criteria of less than 20% mortality/immobilization in all the trials. The control and dispersant only control treatments each showed a very consistent lack of immobilization response across all trials. The results from each trial are visually summarized in the Supplemental Information (SI Fig. 3). The immobilization data from each batch were separately fit against the TPH concentration for each the WAF and CEWAF exposures, as well as combining the responses for each exposure type. Comparing the best fitted model for the TPH-based effective concentration for different experimental trials supported the Weibull1.3 model as the best fitted model in most cases (Table 3). The best fitted model was then used to calculate the EC50 values and 95% confidence intervals.

**Table 3:** The best fitted model based on the AIC criterion and calculated 24-h EC50 values (mg/L) with lower and upper limits (95% confidence limits) calculated on the basis of WAF and CEWAF alone and combined. The lowest EC50 value in each column is bolded and the highest values are bold and italicized

Lobster ID	Batch	WAF		CEWAF		Combined	
		Model	24-h EC50 (95% CI) (TPH mg/L)	Model	24-h EC50 (95% CI) (TPH mg/L)	Model	24-h EC50 (95% CI) (TPH mg/L)
2	1	W2.3	3.60(2.60–4.61)	W1.3	4.71(4.71–4.71)	LL.4	4.72(4.49–4.95)
	2	W1.3	2.93(2.91–2.94)	W1.3	2.28(2.26–2.31)	W1.3	2.91(2.56–3.26)
3	1	W1.4	4.07(3.87–4.27)	W1.3	3.24(3.15–3.33)	W1.3	4.08(4.07–4.10)
4	3	W1.3	**2.30**(2.21–2.40)	W1.3	5.46(4.98–5.94)	W1.3	**2.54**(-6.47–11.56)
6	1	W1.3	3.35(3.07–3.64)	W1.3	3.10(3.02–3.19)	W1.3	5.35(1.90–8.79)
8	1	W1.3	2.81(2.80–2.81)	W1.3	5.33(5.33–5.33)	W1.3	4.54(2.94–6.13)
9	1	W1.3	2.83(2.82–2.84)	W1.3	3.44(3.44–3.44)	W1.3	3.19(2.68–3.70)
	2	LL.4	3.15(2.89–3.41)	LL.4	4.53(0.30–8.75)	LL.4	3.10(1.36–4.85)
15	1	W1.3	3.93(3.93–3.94)	W1.4	**2.15**(2.15–2.15)	W1.3	3.90(2.30–5.50)
16	1	W1.3	4.09(3.05–5.12)	W2.3	8.98(6.10–11.86)	W1.3	4.79(1.20–8.39)
	2	W1.3	2.93(2.91–2.94)	W1.3	6.07(6.02–6.12)	W1.3	3.69(1.02–6.37)
	3	W1.3	2.46(2.37–2.54)	W1.3	7.84(7.84–7.85)	W1.3	5.93(3.02–8.84)
17	1	W2.4	2.84(0.33–5.36)	W1.3	5.33(5.33–5.33)	W1.3	3.52(1.38–5.66)
18	1	W1.3	2.58(2.52–2.62)	W1.3	8.06(6.91–9.20)	W1.3	4.77(-1.09–10.63)
	2	W1.3	3.45(3.17–3.73)	W1.3	7.33(7.15–7.51)	W1.3	3.03(1.60–4.46)
19	1	LL.4	3.04(2.99–3.08)	LL.4	4.37(4.37–4.37)	W1.3	3.15(-3.25–9.55)
21	1	LL.4	3.86(3.23–4.50)	W1.3	5.41(3.51–5.31)	W1.3	3.38(-21.29–28.05)
22	1	W1.4	4.07(3.87–4.27)	W1.4	9.54**(9.50–9.58)	W1.3	4.99(4.99–5.00)
25	3	W2.3	4.77*(3.80–5.73)	LL.4	***12.85***(6.46–19.25)	LL.4	***9.73***(-1.16–20.62)

*LL* Log-logistic, *W1* Type 1 weibull, *W2* Type 2 weibull

*Significant difference between 24 h EC50 value of WAF with CEWAF and Combined (*p* < 0.05

**Significant difference between 24 h EC50 value of CEWAF with WAF and Combined (*p* < 0.05)

**Fig. 2:**
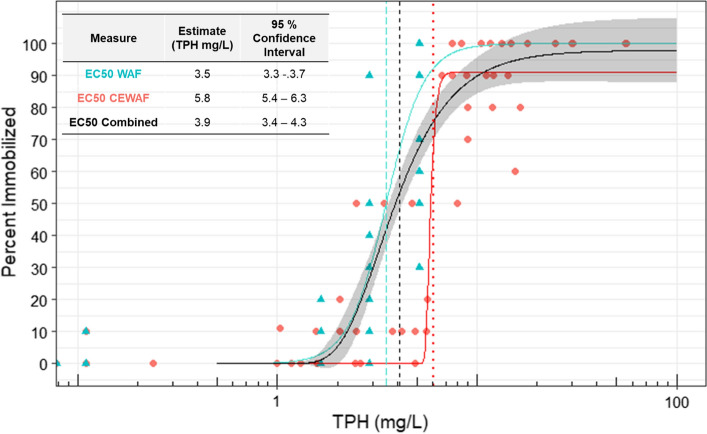
Concentration immobilization response models considering the WAF (blue triangles), CEWAF (red circles), and combined (black line) data. The dashed vertical lines are the EC50 values, which are reported in the inset

**Fig. 3:**
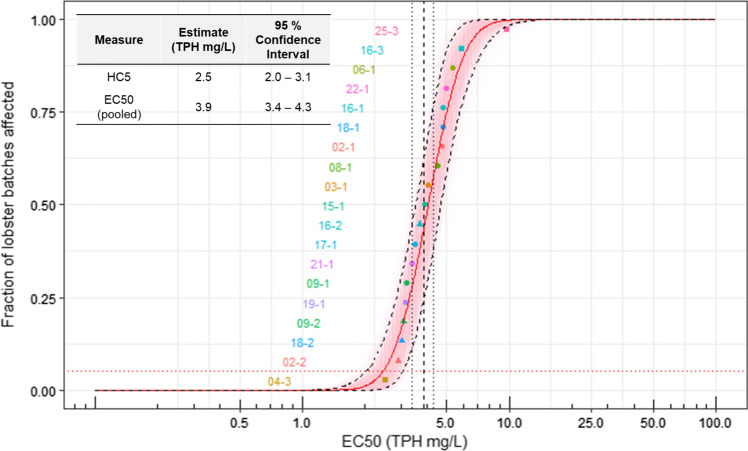
Distribution of lobster toxicity values where each point is an individual batch (lobster ID – release number). The horizontal, dotted, red line is the HC5 estimate of 2.5 mg/L and the vertical dashed black line is the pooled EC50 estimate of 3.9 mg/L

The TPH-based 24-h EC50 values ranged from 2.30 to 4.77 mg/L for the WAF exposures, 2.15–12.8 mg/L for the CEWAF exposures, and 2.54–9.73 mg/L when the exposures were considered together. In each case, the least sensitive batch was the third release from female 25. The most sensitive batch was the first release from female 15 when considering the CEWAF-only exposure, but the third release from female 4 when considering the WAF only and the combined data. There were few differences between the EC50 estimates from a given batch regardless of the method of calculating the effect concentration. This was also seen when the data from all trials were pooled and analysed based on WAF and CEWAF exposures alone and combined (Fig. 2). The EC50 values calculated from the combined responses (Table 3) were used to generate a cumulative distribution from which an HC5 equal to 2.52 mg/L was calculated for exposure to hydrocarbons for larval lobsters (Fig. 3).

### Variability

**Fig. 4:**
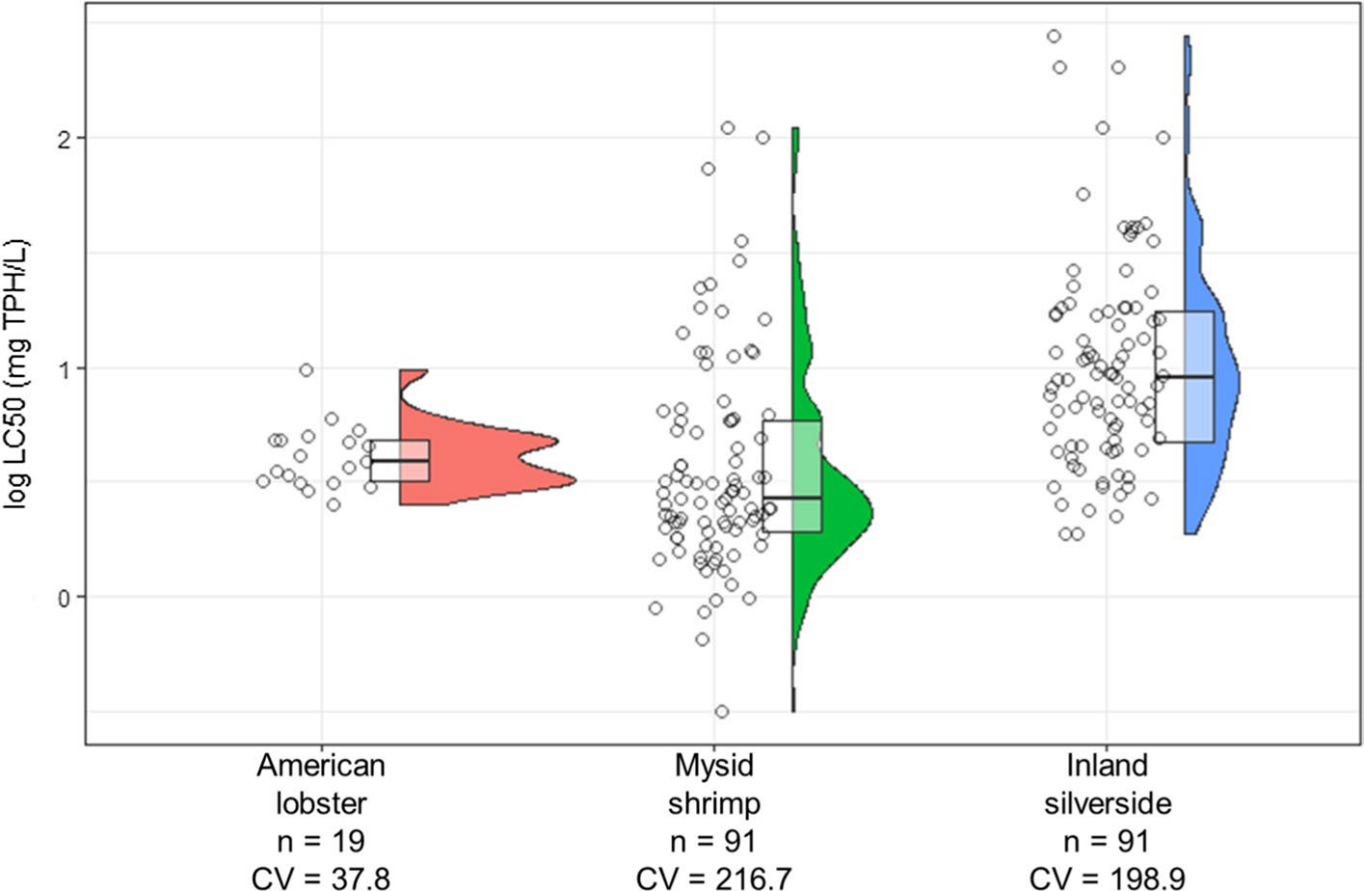
Comparing the variability of 24 h EC50 of American lobster larvae (Homarus americanus) (*n* = 19) exposed to offshore crude oil with the 48-h EC50 of mysid shrimp (Americamysis bahia) (*n* = 91) and 96-h LC50 of inland silversides (Menidia beryllina) (*n* = 91) exposed to No. 2 Fuel Oil (data collected from USEPA NCP Schedule J listings)

The inter-trial variability between the immobilization response of lobster larvae to the total petroleum hydrocarbons was assessed using EC50 values from the combined responses. The coefficient of variation (CV) between all trials was 37%, while that within lobster CV was 23.3% as determined from lobster 16 which had three batches tested, with no significant (*p* > 0.05) difference in response between successive larval batches. The inter-trial variability of the 24-h EC50 values was compared with the acute toxicity of standard test species of mysid shrimp (*Americamysis bahia*) and inland silversides (*Menidia beryllina*) obtained from the US EPA NCP Product Schedule (US EPA 2019). The coefficient of variation was 216.7 and 198.9% for mysid shrimp and inland silverside, respectively (Fig. 4).

## Discussion

The use of reference toxicants allows researchers to provide a measure of precision and proficiency. The use of larval lobsters presents a challenge for a standard reference toxicant quality monitoring programme due to their seasonal availability. However, it is still possible to include reference toxicant testing within a toxicology programme, even if only for a limited season. Our results demonstrated that the bioassay was sensitive, repeatable, and precise, as the achieved LC50 values for copper sulphate align well with what has previously been observed. The results from the reference toxicant testing highlight that the data generated under these testing conditions are reflective of legitimate sensitivity and not an artefact of poor testing conditions, organism health, or handling stress. Based on a curated dataset from the USEPA ECOTOX database (2019), the 48-h LC50 derived in this study places the lobster from this bioassay in the lower 35th percentile of larval marine crustaceans for copper sensitivity (Supplemental Information, Table 3).

Two aspects should be identified to account for the variability of species sensitivity in ecotoxicology: the potential causes of the variability and the significance of those variabilities (Calow 1996). In this study, a consistent methodology was followed to limit the number of variables that may contribute to variability in response. For example, the water quality parameters in the test solutions are considered as potential causes of experimental variabilities (Hrovat et al. 2009), and in this study, water quality parameters were consistent across all trials. Therefore, any variability in response observed in this study would be attributable to biological factors.

Lack of variation in sensitivity of early life stages of lobster to acute exposure of oil may be attributed to narcotic mechanism of action of oil products that is not taxa specific (Barron et al. 2004; Russom et al. 1997). The relatively low variability in the toxic response of lobster larvae might be due to reduced variation in the ability of crustacea to metabolize xenobiotics. There is no consensus on the presence and inducibility of cytochrome 1A (CYP1A) enzymes in invertebrates (Koening et al. 2012). James and Boyle (1998) reviewed cytochrome P450 in crustacea and found evidence that lobsters (*Panulirus argus* and *H. americanus*) do not perform biotransformation of benzo-a-pyrene (B[a]P) in a CYP1A-dependent manner. Most remaining B[a]P was untransformed accumulating in the muscle and hepatopancreas of the American lobster. The authors also noted that the aryl hydrocarbon receptor (AhR) is absent in American lobster. Altogether, it suggests that the lobster does not have the same ability to metabolize and ameliorate exposure to petroleum hydrocarbons as do fish or mammals. The absence, or reduced capacity, for xenobiotic metabolism is compounded by the potentially relatively low level of genetic variability in lobsters, which limits the ability for selection and adaptation. Tracey et al. (1975) employed starch-gel electrophoresis followed by a selective enzyme assay to quantify the amount of genetic variation in geographically distinct (three offshore and five nearshore) natural populations of the American lobster. They found there to be rather low levels of genetic variability within the 300 animals surveyed, with the average proportion of heterozygous loci per individual being 3.8%. The small and nearly constant variance in EC50 values observed for lobsters in this study may be indicative of low phenotypic variability, which can be associated with lower genetic variability (Devin et al. 2014), and may be a reflection of the fact that these wild lobsters all had been caught within the same area (Lobster Fishing Area 36) and are of unknown relatedness. The significance of low variability in terms of sensitivity to hydrocarbons observed in this study may speak to a reduced resilience within this population and a reduced pool of survivors to repatriate following an oil spill.

The procedure used to prepare the exposure medium for toxicity tests can alter the sensitivity of test species to the oil/dispersed oil, irrespective of type of dispersant or oil product. Therefore, determining the concentration of oil constituents is required to account for the subsequent variability in the observed toxic responses, and not just a nominal reporting of dilution. In the current study, combining the WAF and CEWAF exposures along the measured concentration continuum exhibited nearly equal precision when estimating the oil toxicity to lobster larvae than when the WAF and CEWAF were considered alone. The CV of the EC50 values calculated from the combined WAF and CEWAF was equal to 37.8% as compared with the WAF and CEWAF alone with CVs of 20.3% and 47.5%, respectively.

Previous studies have set acceptable ranges for intra- and inter-laboratory variability based on the CV, corresponding to excellent (CV < 35%), good (35–60%), and unacceptable (> 86%) (Echols et al. 2015). The precision in this study puts the larval lobster bioassay in the ‘good’ category and strengthens the reliability of the results. The 24-h LC50 values from this study are in the low mg/L TPH range (mean = 4.2 mg/L), which is consistent with those obtained using the standard test species mysid shrimp (mean = 21.2 mg/L, Fig. 4). The lobster and mysid studies were conducted under different conditions, with different oils, and different durations, and as such a direct comparison is not appropriate. However, the results highlight that the lobster bioassay presented here has similar desirable traits of sensitivity and reproducibility that make it a viable option when there is a desired to perform toxicity testing with local species, such as in a risk or damage assessment.

The difference between the pooled LC50 (3.9 mg/L TPH) and the HC5 (2.5 mg/L TPH) is quite narrow and reflects that an increase in concentration above the HC5 will quickly translate into significant effects. When considering these values for risk assessment, application factors (e.g. 10) could be applied to the HC5 estimate to add a larger degree of conservatism; however, the exposure conditions in this study (static, non-renewal, 24-h duration) likely overestimate the real-world dynamic exposure scenario following an oil spill and thus may provide sufficient conservatism. These results demonstrate that the larval lobster bioassay is sensitive, reliable and may be successfully implemented to assess the hazard from marine contaminants. Application of this bioassay to test the hazard of physically and chemically dispersed crude oil revealed that lobster larvae are sensitive to petroleum hydrocarbon exposure and as a population they exhibit limited variability in response. These observations may place them at greater risk of extirpation should a severe oil spill occur during the same seasonal window when stage I lobster larvae are present.

## Supplementary Information

Below is the link to the electronic supplementary material.

Supplementary file1 (DOCX 502 KB)

## Data Availability

Upon request.

## Code Availability

Upon request.
