# Supplementary material for: Newly Hatched Stage I American Lobster (Homarus americanus) Survival Following Exposure to Physically and Chemically Dispersed Crude Oil
Source: Arch Environ Contam Toxicol. 2022 Jan 27;82(3):307–16. doi: 10.1007/s00244-022-00912-z (PMC8971184; doi:10.1007/s00244-022-00912-z)
Supplement: Supplementary file 1 — Supplementary file1 (DOCX 502 KB) [file 244_2022_912_MOESM1_ESM.docx]

# Supplemental Information

SI Table 1: Overview of the 19 trials conducted with 14 different lobsters. In total there were 30 lobsters collected in 2018, however only 14 lobsters were used in testing. The lobster ID reflects the total number of lobsters available that year, and does not imply sequential order of testing or that some lobsters were skipped.

| **Lobster ID** | **Adult Carapace Length (cm)** | **Wet Weight (g)** | **Estimated volume of egg mass (mm^3^)** | **Batch** | **Date Tested** | **HMSC Trial Code** |
| --- | --- | --- | --- | --- | --- | --- |
| 2 | 9.0 | 729.2 | 36352.3 | 1 | 2018-07-18 | HA-026 |
|  |  |  |  | 2 | 2018-07-19 | HA-029 |
| 3 | 9.8 | 935.9 | 79033.4 | 1 | 2018-07-19 | HA-031 |
| 4 | 9.1 | 686.9 | 18026.3 | 3 | 2018-17-18 | MPRI-HA-003 |
| 6 | 9.9 | 962.1 | 105779.1 | 1 | 2018-07-07 | HA-021 |
| 8 | 10.0 | 962.4 | 84846.2 | 1 | 2018-07-24 | HA-032 |
| 9 | 9.3 | 778.5 | 60074.3 | 1 | 2018-07-18 | HA-027 |
|  |  |  |  | 2 | 2018-07-19 | HA-028 |
| 15 | 10.6 | 1096.7 | 72389.9 | 1 | 2018-07-27 | HA-035 |
| 16 | 10.2 | 1053.0 | 93555.9 | 1 | 2018-07-15 | HA-023 |
|  |  |  |  | 2 | 2018-07-16 | HA-024 |
|  |  |  |  | 3 | 2018-07-17 | HA-025 |
| 17 | 9.1 | 722.9 | 35733.6 | 1 | 2018-07-24 | HA-033 |
| 18 | 9.9 | 910.8 | 76328.8 | 1 | 2018-06-25 | HA-015 |
|  |  |  |  | 2 | 2018-06-26 | HA-017 |
| 19 | 9.6 | 918.9 | 63714.5 | 1 | 2018-07-19 | HA-030 |
| 21 | 8.7 | 666.2 | 30019.1 | 1 | 2018-07-27 | HA-034 |
| 22 | 10.3 | 938.4 | 64392.3 | 1 | 2018-09-13 | MPRI-HA-002 |
| 25 | 10.1 | 1036.0 | 69948.9 | 3 | 2018-09-07 | MPRI-HA-001 |

SI Table 2: The inter-trial coefficient of variation (CV) of water quality parameters (dissolved oxygen, pH, and temperature) at the pre- and post-toxicity test measurements for the CEWAF and WAF treatments. Salinity in all trials ranged from 29 to 32 PSU.

| Treatment | Pre-exposure DO (%) | | | Post-exposure DO (%) | | | |
| --- | --- | --- | --- | --- | --- | --- | --- |
|  | Mean | STD | CV% | Mean | STD | CV% | |
| Control | 94.81 | 2.03 | 2.1 | 83.22 | 7.80 | 9.4 | |
| Control Corexit | 92.66 | 1.73 | 1.9 | 84.30 | 10.86 | 12.9 | |
| WAF 10% | 92.84 | 2.19 | 2.4 | 79.07 | 9.45 | 12.0 | |
| WAF 32% | 92.91 | 2.24 | 2.4 | 80.85 | 8.46 | 10.5 | |
| WAF 100% | 93.22 | 2.12 | 2.3 | 82.77 | 9.72 | 11.7 | |
| CEWAF 1% | 94.89 | 2.36 | 2.5 | 85.89 | 7.64 | 2.9 | |
| CEWAF 3.2% | 94.51 | 2.00 | 2.1 | 80.20 | 7.14 | 8.9 | |
| CEWAF 10% | 94.29 | 2.19 | 2.3 | 82.22 | 7.15 | 8.7 | |
| Treatment | Pre-exposure pH | | | Post-exposure pH | | | |
|  | Mean | STD | CV% | Mean | STD | CV% | |
| Control | 7.74 | 0.13 | 1.7 | 7.72 | 0.22 | 2.8 | |
| Control Corexit | 7.77 | 0.11 | 1.5 | 7.74 | 0.14 | 1.8 | |
| WAF 10% | 7.76 | 0.13 | 1.6 | 7.76 | 0.16 | 2.0 | |
| WAF 32% | 7.77 | 0.12 | 1.6 | 7.75 | 0.15 | 2.0 | |
| WAF 100% | 7.77 | 0.13 | 1.6 | 7.76 | 0.13 | 1.7 | |
| CEWAF 1% | 7.77 | 0.12 | 1.6 | 7.73 | 0.15 | 1.9 | |
| CEWAF 3.2% | 7.77 | 0.12 | 1.5 | 7.74 | 0.11 | 1.5 | |
| CEWAF 10% | 7.78 | 0.12 | 1.5 | 7.76 | 0.12 | 1.5 | |
| Treatment | Pre-exposure Temperature (^o^C) | | | Post-exposure Temperature (^o^C) | | | |
|  | Mean | STD | CV% | Mean | STD | CV% | |
| Control | 14.5 | 0.54 | 3.7 | 14.5 | 0.48 | 3.3 | |
| Control Corexit | 14.5 | 0.58 | 4.0 | 14.5 | 0.44 | 3.0 | |
| WAF 10% | 14.6 | 0.69 | 4.7 | 14.5 | 0.49 | 3.3 | |
| WAF 32% | 14.6 | 0.69 | 4.7 | 14.5 | 0.50 | 3.4 | |
| WAF 100% | 14.7 | 0.74 | 5.1 | 14.6 | 0.49 | 3.4 | |
| CEWAF 1% | 14.5 | 0.59 | 4.1 | 14.6 | 0.41 | 2.8 | |
| CEWAF 3.2% | 14.4 | 0.60 | 4.2 | 14.6 | 0.46 | 3.2 | |
| CEWAF 10% | 14.5 | 0.59 | 4.1 | 14.6 | 0.47 | 3.2 | |
|  |  |  |  |  |  |  |  |

SI Table 3: Chemical characterization of the total petroleum hydrocarbon (TPH) of nominal concentrations of physically dispersed oil (32% WAF) and chemically enhanced fraction of oil (1%, 3.2% and 10% CEWAF) dispersed using Corexit 9500A.

| **Analytes** | **Unit** | **MDL** | **Control Seawater** | **Control Corexit 9500A** | **32% WAF** | **1.0% CEWAF** | **3.2% CEWAF** | **10% CEWAF** |
| --- | --- | --- | --- | --- | --- | --- | --- | --- |
| Naphthalene | µg/L | 0.05 | < MDL | < MDL | 47 | 3.7 | 9.2 | 28 |
| Acenaphthylene | µg/L | 0.01 | < MDL | < MDL | 0.08 | 0.04 | 0.12 | 0.31 |
| Acenaphthene | µg/L | 0.01 | < MDL | < MDL | 0.18 | 0.09 | 0.22 | 0.42 |
| Fluorene | µg/L | 0.01 | < MDL | < MDL | 1.1 | 0.55 | 1.5 | 4 |
| Phenanthrene | µg/L | 0.01 | < MDL | < MDL | 1 | 1.1 | 3.2 | 8.8 |
| Anthracene | µg/L | 0.01 | < MDL | < MDL | < MDL | < MDL | < MDL | < MDL |
| Fluoranthene | µg/L | 0.01 | < MDL | < MDL | < MDL | < MDL | 0.08 | 0.16 |
| Pyrene | µg/L | 0.01 | < MDL | < MDL | < MDL | 0.04 | 0.12 | 0.29 |
| Bz(a)anthracene | µg/L | 0.01 | < MDL | < MDL | < MDL | < MDL | < MDL | < MDL |
| Chrysene/Triphenylene | µg/L | 0.01 | < MDL | < MDL | < MDL | 0.06 | 0.25 | 0.55 |
| Bz(b)fluoranthene | µg/L | 0.01 | < MDL | < MDL | < MDL | 0.02 | 0.07 | 0.19 |
| Bz(k)fluoranthene | µg/L | 0.01 | < MDL | < MDL | < MDL | < MDL | < MDL | < MDL |
| Bz(e)pyrene | µg/L | 0.01 | < MDL | < MDL | < MDL | 0.04 | 0.12 | 0.3 |
| Bz(a)pyrene | µg/L | 0.01 | < MDL | < MDL | < MDL | < MDL | < MDL | 0.04 |
| Indenopyrene | µg/L | 0.01 | < MDL | < MDL | < MDL | < MDL | < MDL | 0.04 |
| Bz(g,h,i)perylene | µg/L | 0.01 | < MDL | < MDL | < MDL | < MDL | < MDL | 0.08 |
| Dibz(a,h)anthracene | µg/L | 0.01 | < MDL | < MDL | < MDL | < MDL | < MDL | < MDL |
| C1-Naphthalenes | µg/L | 0.1 | < MDL | < MDL | 19 | 4.3 | 12 | 37 |
| C2-Naphthalenes | µg/L | 0.1 | < MDL | < MDL | 7.9 | 4.3 | 11 | 36 |
| C3-Naphthalenes | µg/L | 0.1 | < MDL | < MDL | 1.3 | 2.2 | 5.7 | 16 |
| C1-Phenanthrenes | µg/L | 0.1 | < MDL | < MDL | 0.5 | 0.9 | 2.5 | 7.4 |
| C2-Phenanthrenes | µg/L | 0.1 | < MDL | < MDL | 0.4 | 0.9 | 3 | 7.2 |
| C3-Phenanthrenes | µg/L | 0.1 | < MDL | < MDL | < MDL | < MDL | < MDL | < MDL |
| Dibenzothiophene | µg/L | 0.1 | < MDL | < MDL | < MDL | < MDL | < MDL | < MDL |
| C1-Dibenzothiophenes | µg/L | 0.1 | < MDL | < MDL | < MDL | < MDL | < MDL | < MDL |
| C2-Dibenzothiophenes | µg/L | 0.1 | < MDL | < MDL | < MDL | < MDL | < MDL | < MDL |
| C3-Dibenzothiophenes | µg/L | 0.1 | < MDL | < MDL | < MDL | < MDL | < MDL | < MDL |
| 1-methylnaphthalene | µg/L | 0.05 | < MDL | < MDL | 23 | 5.3 | 13 | 40 |
| 2-methylnaphthalene | µg/L | 0.05 | < MDL | < MDL | 20 | 4.7 | 11 | 33 |
| Perylene | µg/L | 0.01 | < MDL | < MDL | < MDL | < MDL | < MDL | < MDL |
| Biphenyl | µg/L | 0.05 | < MDL | < MDL | 3.1 | 0.95 | 2.2 | 7.1 |
| **ΣPAH 31** | µg/L | _ | 0.00 | 0.00 | 124.56 | 29.49 | 75.57 | 227.16 |

**MDL: Method Detection Limit*

*Values ≤ MDL were replaced with* $\frac{1}{2}$ *MDL for calculating sums.*

SI Table 4: Curated results from the USEPA Ecotox database for sensitivity of larval marine crustaceans to copper

| Author | Publication Year | Source | Reference Number | Exposure Type | Species Scientific Name | Species Common Name | Organism Lifestage | 48-hr LC50 (AI mg/L) | Chemical Analysis |
| --- | --- | --- | --- | --- | --- | --- | --- | --- | --- |
| de Jourdan et al. | 2021 | This study |  | Static | Homarus americanus | American Lobster | Larva | 0.123 | Measured |
| McLeese,D.W. | 1976 | Manuscr.Rep.Ser.No.1384, Fish.Res.Board of Can.,Environ.Can., St.Andrews, New Brunswick, Canada:15 p. | 82412 | Renewal | Homarus americanus | American Lobster | Larva | 0.12 | Measured |
| Espiritu,E.Q., C.R. Janssen, and G. Persoone | 1995 | Environ. Toxicol. Water Qual.10:25-34 | 16031 | Static | Artemia sp. | Brine Shrimp | Multiple | 1.3 | Unmeasured |
|  |  |  |  |  |  |  |  | 1.4 | Unmeasured |
|  |  |  |  |  |  |  |  | 2.1 | Unmeasured |
| Xu,Z., L. Hong, and B. Zheng | 1994 | J. Oceanogr. Taiwan Strait (Taiwan Haixia)13(4): 381-387 | 19318 | Not reported | Artemia sp. | Brine Shrimp | Larva | 2.19 | Not reported |
| Espiritu,E.Q., C.R. Janssen, and G. Persoone | 1995 | Environ. Toxicol. Water Qual.10:25-34 | 16031 | Static | Artemia sp. | Brine Shrimp | Multiple | 2.3 | Unmeasured |
|  |  |  |  |  |  |  |  | 3 | Unmeasured |
|  |  |  |  |  |  |  |  | 3.4 | Unmeasured |
| Gajbhiye,S.N., and R. Hirota | 1990 | J. Indian Fish. Assoc.20:43-50 | 17792 | Static | Artemia sp. | Brine Shrimp | Nauplii | 4 | Unmeasured |
| Moraitou-Apostolopoulou,M., and G. Verriopoulos | 1986 | FAO Fish. Rep.334(suppl.): 95-109 | 14474 | Static | Tisbe holothuriae | Harpacticoid Copepod | Nauplii | 0.3142 | Unmeasured |
| Verriopoulos,G., and M. Moraitou-Apostolopoulou | 1982 | Mar. Pollut. Bull.13(4): 123-125 | 11097 | Static | Tisbe holothuriae | Harpacticoid Copepod | Nauplii | 0.3142 | Unmeasured |
|  |  |  |  |  |  |  |  | 0.3415 | Unmeasured |
| Moraitou-Apostolopoulou,M., and G. Verriopoulos | 1986 | FAO Fish. Rep.334(suppl.): 95-109 | 14474 | Static | Tisbe holothuriae | Harpacticoid Copepod | Nauplii | 0.3415 | Unmeasured |
| Verriopoulos,G., and M. Moraitou-Apostolopoulou | 1982 | Mar. Pollut. Bull.13(4): 123-125 | 11097 | Static | Tisbe holothuriae | Harpacticoid Copepod | Copepodid | 0.5289 | Unmeasured |
| Bambang,Y., P. Thuet, M. Charmantier-Daures, J.P. Trilles, and G. Charmantier | 1995 | Aquat. Toxicol.33(2): 125-139 | 16111 | Static | Penaeus japonicus | Kuruma Shrimp | Nauplii | 0.001 | Unmeasured |
|  |  |  |  |  |  |  | Zoea | 0.003 | Unmeasured |
|  |  |  |  |  |  |  | Zoea | 0.02 | Unmeasured |
|  |  |  |  |  |  |  | Zoea | 0.05 | Unmeasured |
|  |  |  |  |  |  |  | Mysis | 0.055 | Unmeasured |
|  |  |  |  |  |  |  | Mysis | 0.06 | Unmeasured |
|  |  |  |  |  |  |  | Mysis | 0.075 | Unmeasured |
|  |  |  |  |  |  |  | Post-larva | 0.11 | Unmeasured |
|  |  |  |  |  |  |  | Juvenile | 1.77 | Unmeasured |
|  |  |  |  |  |  |  | Post-larva | 1.95 | Unmeasured |
|  |  |  |  |  |  |  | Juvenile | 2.5 | Unmeasured |
| Xu,Z., L. Hong, and B. Zheng | 1994 | J. Oceanogr. Taiwan Strait (Taiwan Haixia)13(4): 381-387 | 19318 | Not reported | Fenneropenaeus penicillatus | Redtail Prawn | Larva | 0.016 | Not reported |
| Gao,S., and D. Zou | 1994 | Mar. Sci. Bull. (Haiyang-Tongbao Shuangyuekan)13(2): 28-32 | 16613 | Renewal | Fenneropenaeus penicillatus | Redtail Prawn | Larva | 0.0236 | Unmeasured |
|  |  |  |  |  |  |  | Larva | 0.0389 | Unmeasured |
|  |  |  |  |  |  |  | Post-larva | 1.161 | Unmeasured |
| Lin,S.J., and Y.Y. Tin | 1993 | J. Taiwan Fish. Res.1(2): 55-65 | 14402 | Renewal | Fenneropenaeus penicillatus | Redtail Prawn | Juvenile | 1.3 | Not reported |
|  |  |  |  |  |  |  |  | 1.78 | Not reported |
|  |  |  |  |  |  |  |  | 1.94 | Not reported |
|  |  |  |  |  |  |  |  | 2.31 | Not reported |
|  |  |  |  |  |  |  |  | 3.65 | Not reported |

Supplemental Figures


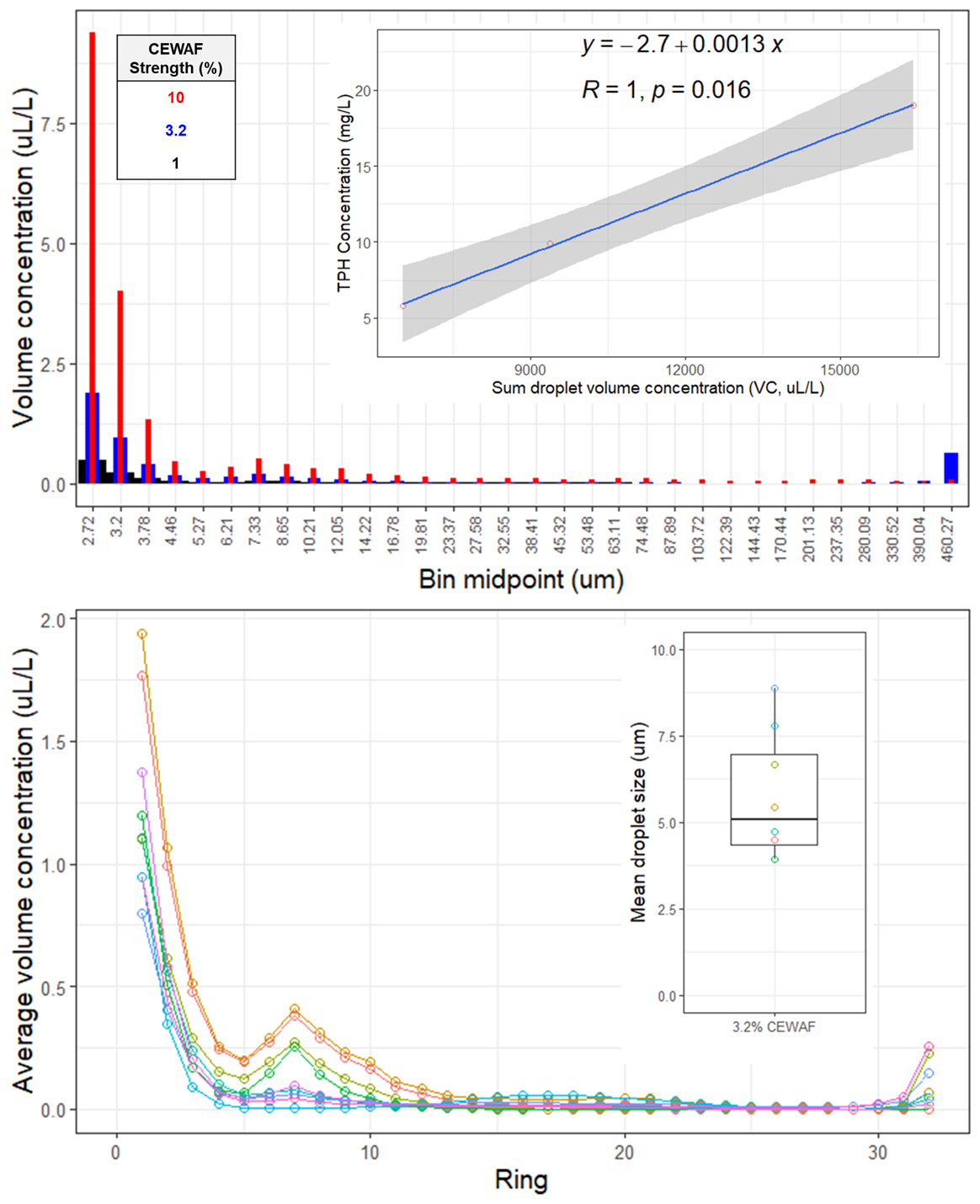


SI Figure 1: (Top) Volume concentration profile by LISST ring midpoint (µm) from a representative 1 (black bars), 3.2 (blue bars), and 10% (red bars) strength CEWAF, with the sum concentration regressed against measured TPH (mg/L) in the insert. (Bottom) Droplet concentration profiles by LISST ring number from nine different preparations of 3.2% strength CEWAF solution, with the mean droplet size (µm) from each preparation in the insert.


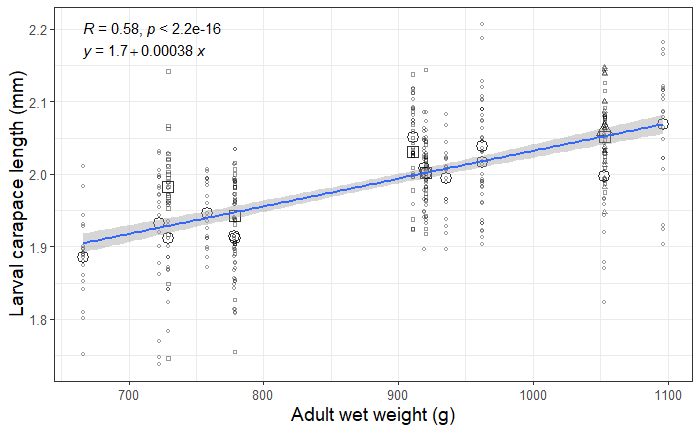


SI Figure 2: The average (larger points) carapace length (mm) of reference lobster larvae (n = 20, smaller points) for each female and release (circle is first release, square is second release, and triangle is third) and the wet weight (g) of each adult lobster.


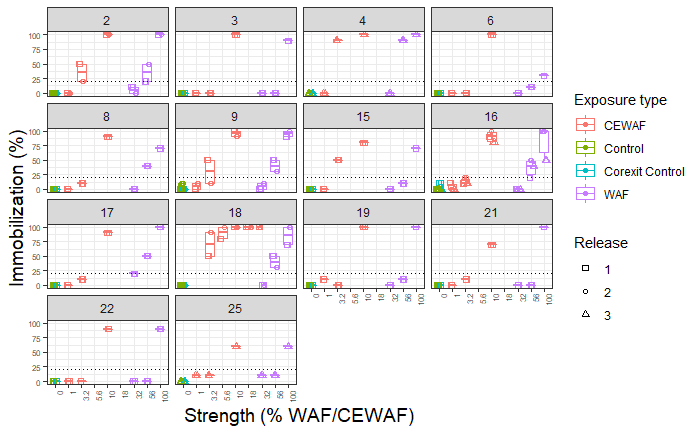


SI Figure 3: Visual summary of the 24-hr immobilization results for larvae from each lobster (individual panels) exposed to dilutions of WAF (purple) and CEWAF (red). The dotted horizontal line is the validity criteria of <20% immobilization in the controls.
